# The Effects of Acute Dopamine Precursor Depletion on the Cognitive Control Functions of Performance Monitoring and Conflict Processing: An Event-Related Potential (ERP) Study

**DOI:** 10.1371/journal.pone.0140770

**Published:** 2015-10-22

**Authors:** Michael J. Larson, Peter E. Clayson, Mark Primosch, Marco Leyton, Scott C. Steffensen

**Affiliations:** 1 Department of Psychology, Brigham Young University, Provo, Utah, United States of America, 84602; 2 Neuroscience Center, Brigham Young University, Provo, Utah, United States of America, 84602; 3 Department of Psychology, University of California Los Angeles, Los Angeles, California, United States of America, 90095; 4 Department of Psychiatry, McGill University, 1033 Pine Ave. W., Montreal, QC, Canada, H3A 1A1; University of Western Ontario, CANADA

## Abstract

Studies using medications and psychiatric populations implicate dopamine in cognitive control and performance monitoring processes. However, side effects associated with medication or studying psychiatric groups may confound the relationship between dopamine and cognitive control. To circumvent such possibilities, we utilized a randomized, double-blind, placebo-controlled, within-subjects design wherein participants were administered a nutritionally-balanced amino acid mixture (BAL) and an amino acid mixture deficient in the dopamine precursors tyrosine (TYR) and phenylalanine (PHE) on two separate occasions. Order of sessions was randomly assigned. Cognitive control and performance monitoring were assessed using response times (RT), error rates, the N450, an event-related potential (ERP) index of conflict monitoring, the conflict slow potential (conflict SP), an ERP index of conflict resolution, and the error-related negativity (ERN) and error positivity (Pe), ERPs associated with performance monitoring. Participants were twelve males who completed a Stroop color-word task while ERPs were collected four hours following acute PHE and TYR depletion (APTD) or balanced (BAL) mixture ingestion in two separate sessions. N450 and conflict SP ERP amplitudes significantly differentiated congruent from incongruent trials, but did not differ as a function of APTD or BAL mixture ingestion. Similarly, ERN and Pe amplitudes showed significant differences between error and correct trials that were not different between APTD and BAL conditions. Findings indicate that acute dopamine precursor depletion does not significantly alter cognitive control and performance monitoring ERPs. Current results do not preclude the role of dopamine in these processes, but suggest that multiple methods for dopamine-related hypothesis testing are needed.

## Introduction

Cognitive control refers to the monitoring, planning and regulation of effective, situation-appropriate goal-directed behaviors. Mesocorticolimbic dopamine pathways play a prominent role in these processes [1]. Findings bolstering a relationship between dopamine and cognitive control in humans are anchored in studies of healthy controls taking dopamine-related medications [2, 3, 4] or studies of individuals with disorders with known dopamine irregularities such as schizophrenia [1, 5]. However, non-specific pharmacological effects or disease-related processes confound the results of such studies [6–8]. For example, treatments known to enhance dopamine transmission may improve some aspects of cognition, such as task switching, while adversely affecting other processes, such as learning from negative feedback [7]. We assessed the effects of acute dopamine amino acid (AA) precursor depletion on cognitive control processing in drug-free participants without the use of a neuropsychiatric medication.

The acute phenylalanine (PHE) and tyrosine (TYR) depletion (APTD) method rapidly decreases dopamine transmission with few side effects [9–12]. Participants ingest a protein mixture deficient in the essential amino acid PHE and its hydroxylated product, TYR, the AA substrate for the rate-limiting enzyme in dopamine synthesis, tyrosine hydroxylase (TH). Since TH is normally incompletely saturated, dopamine synthesis in the brain is dependent upon TYR availability. APTD treatments, therefore, lead to marked reductions in PHE and TYR availability, reducing dopamine synthesis and release in the striatum and areas of the cortex, including the anterior cingulate cortex (ACC) [10, 12, 13, 14, 15].

Multiple cognitive control functions can be effectively measured using event-related potentials (ERPs). Cognitive control-related ERPs are often temporally locked to either conflict-related stimuli, such as in the flanker, Stroop, or Simon task, or are response-locked to examine error-related conflict and performance monitoring (see [16] for review of conflict-related ERPs). From a stimulus-locked perspective, when using the Stroop task, the N450 is a fronto-central negativity in the ERP that peaks at approximately 450 msec and is more negative for incongruent Stroop trials than congruent Stroop trials, suggesting a role in conflict detection [17, 18]. The N450 is also more negative as stimuli increase in level of incongruence or as more conflict-related interference is present, further suggestive of a role in conflict-related processing [17, 19, 20].

The conflict slow potential (also known as the conflict SP or negative slow wave [NSW]) is a sustained centro-parietal positivity that begins at approximately 500 msec that is thought to reflect a signal for increased recruitment of cognitive control resources [18, 21]. A more positive conflict SP is associated with increased response times and lower error rates, potentially indicating a role for the conflict SP in resolution of conflict or selecting an appropriate response [22]. Multiple methods evidence the ACC as the primary neural generator of the N450 [23, 24], whereas the conflict SP is likely generated from sources in the lateral prefrontal and posterior cortices [24, 25].

From a response-locked perspective, the error negativity (Ne; [26]) or error-related negativity (ERN; [27]) and the error positivity (Pe; also referred to as the post-error positivity) [26, 28, 29] represent response-related performance monitoring processes. Specifically, the ERN is a negative deflection in the ERP that peaks approximately 50 msec following error commission and is thought to be generated by the ACC [30–33]. The functional significance of the ERN is a matter of some debate, but is thought to reflect the detection of response conflict [34], an early error detection mechanism [26, 35], or (perhaps most important to the current manuscript) a dopamine-related reinforcement-learning signal [36].

The Pe, in contrast, is a sustained centro-parietal positivity that tends to be maximal between 200ms to 400ms after error commission. The predominant view is that the Pe is an electrophysiological indication of conscious error awareness [18, 37–40] or an affective response to making a mistake [28, 29]. The localization of the Pe is a matter of continued research with reports of contributions from the ACC [30, 41], posterior portions of the cingulate [42], and parietal or insular cortices [43].

Dopamine has been implicated in multiple cognitive control processes related to both stimulus-locked and response-locked cognitive control ERPs. For example, one prominent computational model, the reinforcement learning theory (RL-ERN), focuses on generation of the ERN (and a feedback-related counterpart known as the feedback negativity [FRN]) and suggests these ERPs reflect a reinforcement-learning signal sent by phasic dips in mesencephalic dopamine activity to the ACC that updates the response selection process [36]. The ACC is then thought to integrate the reinforcement history over time in order to optimize the response selection process [44, 45]. Support for the RL-ERN theory comes primarily from studies finding aberrant ERN, CRN, and FRN amplitudes in various neurological conditions associated with dopamine system dysfunction, such as Parkinson’s disease, attention deficit/hyperactivity disorder, and schizophrenia [5, 7, 46–52].

Several studies have examined the relationship between dopamine-related psychoactive medications and ERN amplitude in healthy individuals. For example, dopamine agonists (specifically indirect dopamine agonists) appear to increase ERN amplitude [2, 53, 54], whereas dopamine antagonists, such as haloperidol, tend to decrease ERN amplitude [3, 55]. These findings support a relationship between ERN amplitude and medication-related changes in dopamine functioning in healthy individuals. Notably, the little research that exists suggests dopaminergic manipulation does not influence Pe amplitude [2, 29, 54].

Stimulus-locked conflict processing may also be affected by dopaminergic inputs. Specifically, there is a decline in Stroop performance in individuals with psychiatric and neurologic conditions with dopamine dysregulation, such as schizophrenia or Parkinson’s disease, with studies indicating these deficits are likely related to ACC functioning [1, 56, 57]. In addition, genetic work suggests that individuals with genotypes associated with increased striatal dopamine D1 receptor function and mRNA expression show enhanced N450 amplitudes relative to participants with other D1 genotypes [58, 59]. It is, therefore, possible that dopamine modulation of direct and indirect pathways related to striatal and ACC functioning enhances or decreases N450 amplitudes and related conflict monitoring [59].

There is no direct research that we are aware of on conflict SP amplitudes and direct dopaminergic inputs. However, attentional control seems to be affected by dopamine precursor depletion. For example, Scholes et al. [6] found decreased Stroop interference following APTD. These authors interpreted their findings to suggest improved gating of information by reduced noise in the monoamine system. Thus, there appears to be support for the idea that conflict-related ERPs such as the conflict SP will be affected by procedures such as APTD, although this has not yet been directly tested.

Brain dopamine activity can be indexed through neuroendocrine analysis of plasma prolactin levels. Since dopamine exerts an inhibitory action on prolactin release in the hypothalamus, increased prolactin levels are indicative of a decrease in dopamine transmission [60, 61]. Based on evidence implicating dopamine signaling in ACC-related cognitive control processes and implicating the ACC in N450, ERN, and possibly Pe generation, we hypothesized decreased amplitude N450 and ERN amplitudes during the APTD condition relative to a nutritionally balanced AA mixture (BAL). We did not have specific hypotheses about amplitudes of the Pe and conflict SP as the reliance of these two ERP components on medial frontal structures and dopamine-mediated processes is not yet clear in the current literature.

## Materials and Methods

### Ethics Statement

The Brigham Young University Institutional Review Board approved all study procedures in accord with all principles expressed in the Declaration of Helsinki. All participants provided written informed consent.

### Participants

Initial study enrollment consisted of sixteen males. Three participants did not return for the follow-up session and one participant terminated his first session early due to feeling ill. Final study enrollment, therefore, included twelve males between 18 and 30 years of age (*M* = 22.67, *SD* = 2.74) who completed two sessions in a within-subjects, double-blind design. Considering previously shown sex differences for the effects of acute tryptophan depletion [62] and conflict and cognitive control ERPs [63–65], the study was restricted to males. The procedures for acute dopamine precursor depletion used in this study have been described previously [11]. The day prior to each testing session participants were provided a low-protein diet and fasted from midnight until arriving for the study at 07:00 hours. Upon arrival for the study, participants had blood samples drawn. They were subsequently administered either a nutritionally balanced AA mixture (comparison condition) or an AA mixture deficient in PHE and TYR (depletion condition). Depletion or balanced condition order was decided randomly using a random number generator and scheduled seven days apart. Eight of the twelve participants were randomized to start with the depletion condition; however, order effects did not meaningfully affect the amino acid or prolactin values (or the primary study results) when comparing the different starting conditions and the condition assignment was truly random. After ingesting the mixture, participants were asked to remain awake in a quiet room reading neutral or school-related material. At 11:00 hours a second blood draw was completed to ensure dopamine precursor depletion, followed by the EEG application and Stroop task described below. Additional tasks, including neuropsychological measures of executive functions, color processing, and mood were administered but are not reported here. All tasks were administered in counterbalanced order between 11:00 and 13:00 hours.

Exclusion criteria were assessed via participant self-report and included current or previous diagnosis of a psychiatric disorder, psychoactive medication use, current substance use (including alcohol, illicit drugs, or tobacco—no participant had ever used any of these substances), neurological disorders, head injury, left-handedness, uncorrected visual impairment, or color blindness. Absence of color blindness was ensured using the Ishihara pseudo-isochromatic plate color vision test [66].

### Plasma Amino Acids

Plasma AA levels, except for tryptophan, were measured using high-pressure liquid chromatography with fluorometric detection (HPLC-FD) on an Ultraspher ODS reverse-phase column (Beckman Coulter, Fullerton, CA) with o-phtalaldehyde pre-column derivatization and aminoadipic acid as an internal standard. Total tryptophan levels were measured by HPLC-FD on a Bondpak^®^ reverse-phase column (Phenomenex, Torrance, CA). No measurements were made of catecholamines or their metabolites in plasma because these measures would reflect primarily effects on catecholamine synthesis in the periphery, which would not necessarily be of the same magnitude as those in the brain. TYR availability in the brain, but not in the periphery, is influenced by the levels of the other LNAAs that inhibit the transport of the AA across the blood brain barrier [67].

### Experimental Tasks

Participants completed a modified color-naming version of the Stroop task [68] wherein they were presented with a Stroop stimulus with the words “RED”, “GREEN”, “BLUE”, or “GRAY” written in red, green, blue, or gray colored-font. Congruent trials consisted of words presented in their same color font (e.g., RED printed in red font); incongruent trials consisted of color-words printed in a different color of font (e.g., RED printed in blue font). Participants were instructed to respond as quickly and accurately as possible to the color of the font with a button press to one of four response keys using the index, middle, ring, and pinky fingers of their right hand. Participants were presented ten blocks of 80 trials (800 total trials). There were 600 (75%) congruent trials and 200 (25%) incongruent trials. Stroop stimuli were presented for 1,000 msec. The inter-trial interval consisted of a blank screen that varied randomly from 1,000 msec to 1,500 msec. Color-to-key mapping was practiced prior to task performance using 25 presentations of each color-key combination. Color-to-key mapping was counterbalanced across participants and APTD and BAL conditions.

### Prolactin Levels

Prolactin levels were determined by a two-step Chemiluminescent Microparticle Immunoassay (CMIA; Abbott Architect i2000). In the first step, specimen and anti-prolactin antibody (mouse, monoclonal) coated paramagnetic microparticles were combined. Prolactin present in the specimen was bound to the anti-prolactin (mouse, monoclonal) coated microparticles. After washing, anti-prolactin (mouse, monoclonal) acridinium labeled conjugate was added in the second step. Pre-Trigger and Trigger solutions were then added to the reaction mixture; the resulting chemiluminescent reaction was measured as relative light units (RLUs). A direct relationship exists between the amount of prolactin in the specimen and the RLUs detected by the Architect *i* optical system.

### Electrophysiological Data Recording

Electroencephalographic (EEG) activity was recorded from 128 scalp sites using a Geodesic sensor net and Electrical Geodesics, Inc. (EGI; Eugene, Oregon) amplifier system (20K nominal gain, bandpass = 0.10–100Hz). Electroencephalographic activity was referenced to the vertex electrode and digitized continuously at 250Hz with a 24-bit analog-to-digital converter. Impedances were maintained below 50k consistent with recommendations of the manufacturer. Digitized data were high-pass filtered at 0.1Hz and low-pass filtered at 30Hz.

### Event-related Potential Reduction and Measurement

Eye blinks were removed from the segmented waveforms using independent components analysis (ICA) in the ERP PCA Toolkit [69] that utilizes EEGLAB [70]. The ICA components that correlated at 0.9 with the scalp topography of two blink templates, one generated based on the current data and another provided by the ERP PCA Toolkit author, were removed from the data [71]. Trials were considered “bad” if more than 10% of channels were marked. Channels were marked bad if the fast average amplitude exceeded 100 μV or if the differential average amplitude exceeded 50 μV. Channels were also marked globally bad for the entire session if more than 20% of the trials were deemed bad. To correct bad channels, spline interpolation was performed from good channels. Data were average re-referenced and the polar average reference effect (PARE) was implemented to correct for under-sampling of the undersurface of the head [72].

Individual-subject stimulus-locked congruent and incongruent trials were segmented spanning 250 msec prior to stimulus presentation to 1000 msec after stimulus presentation. Data were baseline adjusted using a 200 msec window from -250 msec to -50 msec before stimulus presentation. Electrode sites and time windows for stimulus-locked ERP analyses were based on previous work and an examination of the present waveforms for the N1 [73, 74], P2 [74, 75], N2 [75, 76], P3 [77, 78], N450 [18, 21, 79], and conflict SP [18, 21, 79]. An adaptive mean approach was implemented to reduce the deleterious effects of background EEG noise while capturing individual-subject variability in peak latencies [80]. The N1, P2, N2, and N450 was extracted as the average of activity at fronto-medial electrode sites (6 [FCz], 7, 106, and 129 [Cz]; see [81] for sensor layout). The P3 and conflict SP was extracted as the average activity at parietal electrode sites (62 [Pz], 67, 71, 72, 76, and 77; see [81]). Amplitude measurements were extracted as the average activity from 15 msec pre-peak to 15 msec post-peak negative amplitude between 75 msec and 175 msec for the N1, between 250 msec and 350 msec for the N2, and between 400 and 500 msec for the N450. The average activity from 15msec pre-peak to 15 msec post-peak positive amplitude between 150 msec and 250 msec for the P2 and 300 msec and 400 msec for the P3. Conflict SP amplitudes were extracted as the mean amplitude from 650 msec to 750 msec post-stimulus presentation. Latency measurements were scored during the same time windows for the N1, P2, N2, P3, and N450. Given the tonic nature of the conflict SP, latency times were not calculated.

Individual-subject, response-locked correct and error trials were segmented spanning 300 msec prior to the response to 800 msec after the response. Data were baseline adjusted using a 200 msec window from -300 msec to -100 msec before the participant’s response. Electrode sites and time windows for response-locked ERP analyses were based on previous work and an examination of the present data for ERN and Pe [27, 28, 29, 82]. The ERN was extracted as the average of activity at fronto-medial electrode sites (FCz, 7, 106, Cz). The Pe was extracted as the average activity at centro-parietal electrode sites (54, 55, 61, 78, 79, Pz). Correct and error trial amplitudes for the ERN were extracted as the average of 15 msec pre-peak to 15 msec post-peak negative amplitude between 0 msec and 150 msec. Latency measurements for the ERN were extracted as the peak negative-going amplitude between 0 msec and 150 msec following the participant’s response averaged across the same fronto-central electrode locations. Correct and incorrect Pe amplitudes were extracted as the mean amplitude from 200 msec to 400 msec post-stimulus presentation. Given the tonic nature of the Pe, latency times were not extracted.

### Data Analysis

Paired-samples t-tests were used to compare plasma levels of amino acids and prolactin plasma levels between the placebo and depletion conditions. In addition to individual analyses of each amino acid, we calculated the tyrosine/large neutral amino acid (LNAA) ratio by dividing the tyrosine amino acid level by the sum of tyrosine + phenylalanine + tryptophan + valine + leucine + isoleucine. Decreases in the tyrosine/LNAA are indicative of diminished availability of tyrosine for entry into the brain [83]. Subsequently, to reduce Type I error and to avoid the biasing effects of non-normality typical of small sample sizes, as well as (co)variance heterogeneity between groups [84], robust analyses of variance (ANOVAs) were conducted using the ERP PCA Toolkit. Robust statistics are more conservative than conventional ANOVAs and help avoid erroneous findings from inflated Type I error rates [69, 85]. The number of iterations used for bootstrapping was 50,000. To further ensure the manipulation altered amino acid levels, separate 2-Condition (APTD, BAL) x 2-Time (Pre [prior to mixture ingestion], Post [four hours following mixture ingestion]) robust ANOVAs were performed on TYR and PHE measurements. To test the study hypotheses we used separate 2-Condition (APTD, BAL) x 2-Congruency (congruent, incongruent) robust ANOVAs on mean response times (RTs), error rates, and stimulus-locked ERP amplitude and latency data. Separate 2-Condition x 2-Accuracy (correct, incorrect) robust ANOVAs were conducted on post-accuracy RTs, ERN and Pe amplitudes. Data for the primary analyses are included as a supplementary file S1 Dataset.

## Results

### Amino Acid Levels

**Fig 1:**
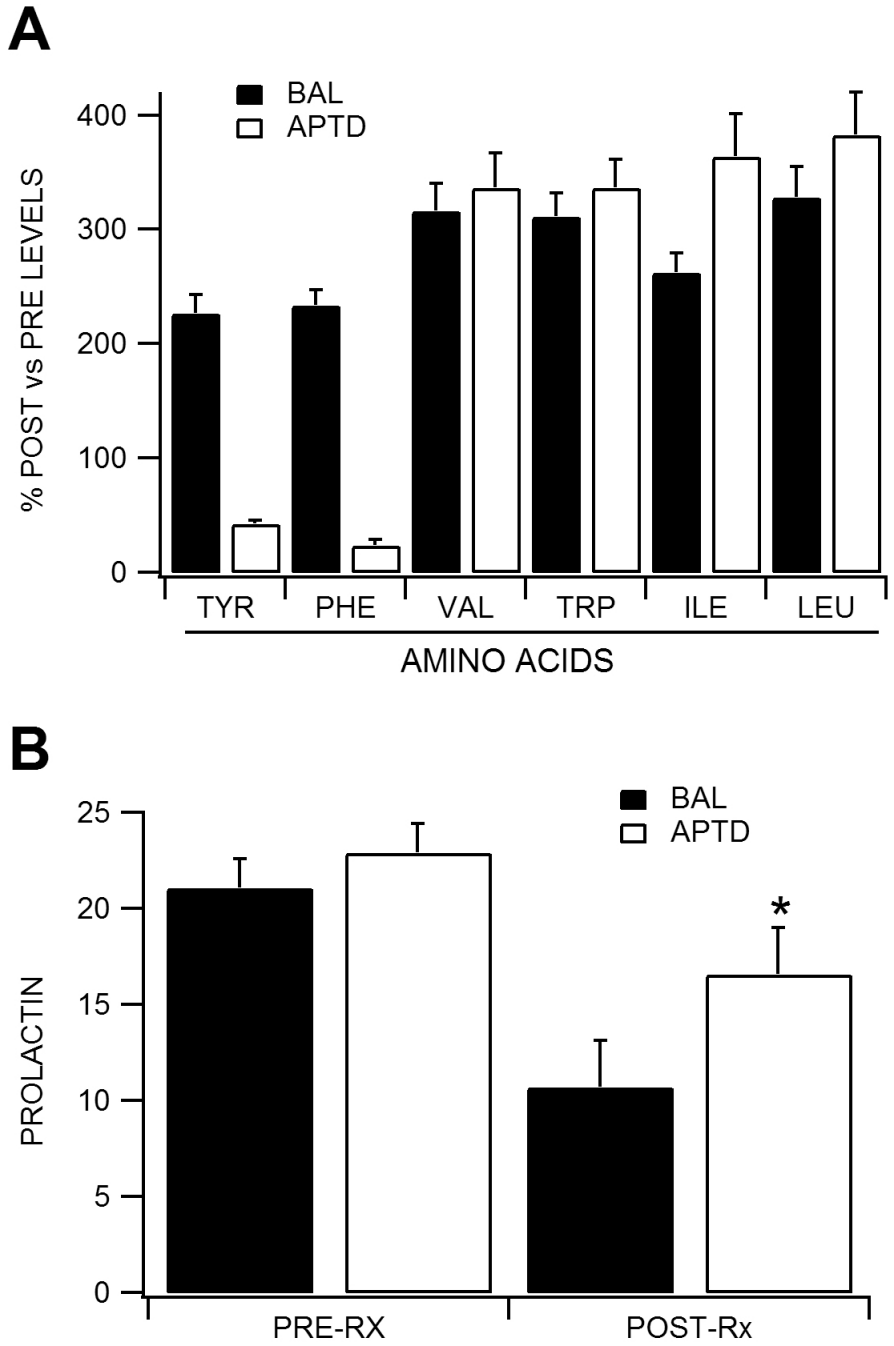
Percentage of baseline A) amino acid and B) prolactin levels during APTD and BAL conditions.

Plasma levels of TYR, PHE and the large neutral AAs valine (VAL), tryptophan (TRP), isoleucine (ILE), and leucine (LEU) were compared between the APTD and BAL treatments. Table 1 shows the plasma levels for each AA pre-ingestion versus post-ingestion of the APTD and BAL mixtures. There was no significant difference in baseline AA levels between APTD and BAL treatments (all *t* values < 1.16, *p* values > .27). BAL treatment resulted in a significant increase in all amino acids from pre-ingestion to post-ingestion (see Fig 1A; TYR: *t*(11) = 5.30, *p* < .001; PHE: *t*(11) = 8.16, *p* < .001; VAL: *t*(11) = 10.90, *p* < .001; TRP: *t*(11) = 8.39, *p* < .001; ILE: *t*(11) = 8.66, *p* < .001; LEU: *t*(11) = 8.56, *p* < .001), while APTD treatment resulted in a specific and significant reduction in TYR and PHE levels and a significant increase in VAL, TRP, ILE and LEU levels (TYR: *t*(11) = -15.36, *p* < .001; PHE: *t*(11) = -12.59, *p* < .001; VAL: *t*(11) = 8.00, *p* < .001; TRP: *t*(11) = 5.39, *p* < .001; ILE: *t*(11) = 6.62, *p* < .001; LEU: *t*(11) = 9.06, *p* < .001). Notably, the tyrosine/LNAA ratio did not differ at pre-ingestion between the APTD and BAL treatments (*t*(11) = 0.38, *p* = .71; APTD: mean ratio = 0.13±.01; BAL: mean ratio = 0.13±.02). The tyrosine/LNAA ratio decreased significantly from pre-ingestion to post-ingestion for both the BAL, *t*(11) = 2.97, *p* = .01, and APTD treatments, *t*(11) = 17.58, *p* < .001; however, comparison of the post-treatment tyrosine/LNAA ratios showed a significantly lower ratio following the APTD relative to the BAL treatment, *t*(11) = 6.63, *p* < .011 (mean post-ingestion ratio for BAL = .10±.04; mean post-ingestion ratio for APTD = .02±.02).

**Table 1:** Summary Data for Plasma Concentration Levels of Amino Acids Before and After Ingesting the Amino Acid Mixture (*n* = 12).

	APTD				BAL			
	Pre		Post		Pre		Post	
	Mean (μmol/l)	SEM	Mean (μmol/l)	SEM	Mean (μmol/l)	SEM	Mean(μmol/l)	SEM
TYR	15.7	0.7	7.0	1.3	14.0	0.7	30.4	3.2
PHE	11.9	1.1	3.4	1.4	10.4	0.6	24.4	2.2
VAL	34.1	3.0	111.4	9.3	31.2	2.4	93.6	6.9
TRP	24.3	2.8	81.8	12.4	23.3	3.0	66.6	7.3
ILE	10.0	0.9	36.9	3.9	9.3	0.7	23.3	1.8
LEU	21.2	1.8	82.0	6.3	19.7	1.4	63.0	5.4

APTD = acute phenylalanine and tyrosine depletion; BAL = balance amino acid mixture; TYR = tyrosine; PHE = phenylalanine; VAL = valine; TRP = tryptophan; ILE = isoleucine; LEU = leucine

The robust ANOVA on TYR plasma concentration indicated a significant Condition x Time interaction, *T*_WJt_/c(1.0,11.0) = 50.43, *p* = .0002. Plasma concentration of TYR decreased significantly during the APTD condition and increased significantly during the BAL condition, *T*_WJt_/c(1.0,11.0) = 235.78, *p*<0.0001; *T*_WJt_/c(1.0,11.0) = 28.04, *p* = .0006, respectively. Plasma concentration was higher for BAL post scores compared to APTD post scores, *T*_WJt_/c(1.0,11.0) = 38.75, *p*<0.0001. For PHE plasma concentration, the Condition x Time interaction was significant, *T*_WJt_/c(1.0,11.0) = 12.04, *p*<0.0001. Plasma concentration of PHE decreased significantly during the APTD condition and increased significantly during the BAL condition, *T*_WJt_/c(1.0,11.0) = 158.62, *p*<0.0001; *T*_WJt_/c(1.0,11.0) = 66.65, *p* = .0003, respectively. Plasma concentration was higher for BAL post scores compared to APTD post scores, *T*_WJt_/c(1.0,11.0) = 55.16, *p* < .0001.

### Prolactin Levels

As a further manipulation check, prolactin levels were also compared using the blood samples taken before and after ingestion of the APTD or BAL mixtures. There was no significant difference in baseline pre-ingestion prolactin levels between APTD and BAL treatments (*t*(11) = 0.87, *p* = .40; APTD: mean prolactin level = 22.5±2.0 ug; BAL: mean prolactin level = 21.3±2.1 ug). Prolactin levels decreased significantly from pre-ingestion to post-ingestion of the BAL treatment (see Fig 1B; *t*(11) = 5.39, *p* < .001; mean post BAL prolactin level = 11.0±0.6 ug), but not following the APTD treatment (*t*(11) = 0.61, *p* = .55; mean post APTD prolactin level = 20.2±3.7 ug). This pattern is consistent with diminished release of dopamine at the hypothalamic level in the APTD condition.

### Response Times and Error Rates

Mean response time (RT), post-accuracy RTs, and error rate data as a function of group are presented in Table 2. Initial tests examined the effect of order of placebo and depletion condition and showed no significant congruency differences as a function of condition order for RTs or error rates (all *p* values > .21). Subsequent tests of study hypotheses indicated there was a significant main effect of congruency with longer RTs for incongruent relative to congruent trials, *T*_WJt_/c(1.0,11.0) = 85.56, *p* < .0001. The main effect of condition and the Condition x Congruency interaction were not significant, *T*_WJt_/c(1.0,11.0) = 0.02, *p* = .88; *T*_WJt_/c(1.0,11.0) = 0.08, *p* = .79, respectively. When examining post-accuracy RTs, the main effect of condition was significant with longer post-error RTs than post-correct RTs, *T*_WJt_/c(1.0,11.0) = 8.69, *p* = .02. The main effect of condition and the Condition x Congruency interaction were not significant, *T*_WJt_/c(1.0,11.0) = 0.27, *p* = .61; *T*_WJt_/c(1.0,11.0) = 0.04, *p* = .84, respectively.

**Table 2:** Demographic and Mean Response Times, Error Rate, and Event-Related Potential Trial Summary Data.

	APTD		BAL	
	Mean	SD	Mean	SD
Congruent RT (msec)	570	54	567	42
Incongruent RT (msec)	636	53	632	42
Post-correct RT (msec)	584	38	589	56
Post-error RT (msec)	602	33	610	47
Congruent error rates	12%	9%	12%	11%
Incongruent error rates	20%	12%	19%	13%
Congruent trials retained	322	112	350	129
Incongruent trials retained	129	47	138	52
Correct trials retained	607	112	507	164
Error trials retained	56	64	42	20

Estimates for behavioral and stimulus-locked measures contain data from 12 males; estimates for response-locked measures contain data from 11 males. Congru ent and incongruent trials retained and correct and error trials retained indicate the number of trials retained for averaging following artifact correction and rejection. APTD = acute phenylalanine and tyrosine depletion; BAL = balance amino acid mixture; RT = response time (in msec)

For error rates, there was a significant main effect of congruency, *T*_WJt_/c(1.0,11.0) = 54.60, *p* < .0001. Error rates were larger for incongruent trials than for congruent trials. Neither the main effect of condition nor the Condition x Congruency interaction was significant, *T*_WJt_/c(1.0,11.0) = 0.10, *p* = .78; *T*_WJt_/c(1.0,11.0) = 0.41, *p* = .55, respectively. In sum, RT and error rate data differed by Stroop task congruency but not as a function of APTD or BAL condition.

### Event-Related Potentials

**Fig 2:**
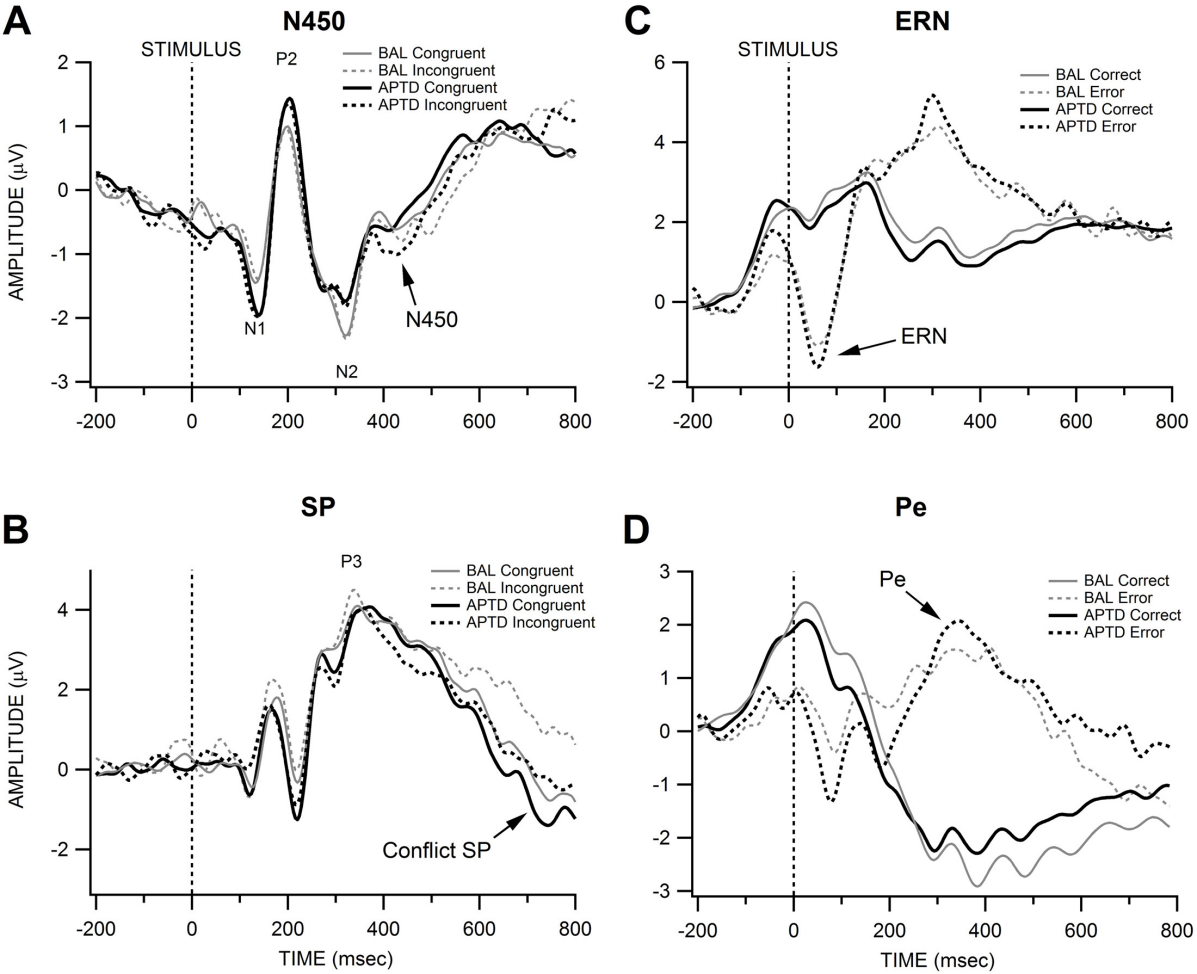
Grand averaged A) N450 and B) conflict slow potential (SP) waveforms of stimulus-locked congruent and incongruent trials averaged across fronto-medial electrode sites for the N450 and parietal electrode sites for the conflict SP. C) error-related negativity (ERN) and D) error positivity (Pe) waveforms of response-locked correct and incorrect trials averaged across fronto-medial electrode sites for the ERN and centro-parietal electrode sites for the Pe.

Grand averaged ERP waveforms as a function of condition are presented in Fig 2; component amplitude and latency data are contained in Table 3, and the numbers of trials retained for averaging are presented in Table 2. Correct-trial, response-locked ERPs contained a minimum number of 199 trials and a maximum number of 743 trials; error-trial, response-locked ERPs contained a minimum number of 9 trials and a maximum number of 231 trials. Congruent, stimulus-locked ERPs contained a minimum number of 101 trials and a maximum number of 504 trials; incongruent, stimulus-locked ERPs contained a minimum number of 41 trials and a maximum number of 198 trials. Noise estimates [80, 86] and number of trials retained for averaging were not significantly different between BAL and APTD conditions (all *T* values < 5.1, all *p* values >.10). Initial analyses of condition order showed no significant order-related differences for any of the ERPs (all p values > .13).

**Table 3:** Event-Related Potential Amplitude (μV) and Latency (msec) Summary Data.

	APTD		BAL	
	Mean	SD	Mean	SD
Congruent N1 amplitude	-2.2	1.3	-1.6	1.1
Incongruent N1 amplitude	-2.3	1.3	-1.6	1.4
Congruent N1 latency	140	20	133	22
Incongruent N1 latency	142	13	124	30
Congruent P2 amplitude	1.6	2.0	1.4	1.8
Incongruent P2 amplitude	1.5	2.0	1.5	1.9
Congruent P2 latency	201	20	199	22
Incongruent P2 latency	201	19	194	21
Congruent N2 amplitude	-2.3	1.5	-2.5	1.4
Incongruent N2 amplitude	-2.2	1.5	-2.6	1.4
Congruent N2 latency	304	28	313	23
Incongruent N2 latency	302	19	313	25
Congruent P3 amplitude	4.6	2.4	4.4	3.0
Incongruent P3 amplitude	4.5	2.3	4.7	2.7
Congruent P3 latency	349	30	353	18
Incongruent P3 latency	352	23	346	14
Congruent N450 amplitude	-1.0	1.5	-.9	1.7
Incongruent N450 amplitude	-1.5	1.6	-1.3	1.8
Congruent N450 latency	447	38	450	31
Incongruent N450 latency	445	33	458	30
Congruent conflict SP amplitude	0.0	2.5	-.4	2.3
Incongruent conflict SP amplitude	1.5	2.3	0.5	2.0
CRN amplitude	1.6	1.5	1.9	1.7
ERN amplitude	-1.9	1.4	-1.4	1.2
CRN latency	54	38	50	46
ERN latency	63	21	68	20
Pc amplitude	-1.8	1.3	-1.9	2.2
Pe amplitude	0.9	1.3	1.0	1.6

Note. Estimates for behavioral and stimulus-locked measures contain data from 12 males; estimates for response-locked measures contain data from 11 males. APTD = acute phenylalanine and tyrosine depletion; BAL = balance amino acid mixture; conflict SP = conflict slow potential; CRN = correct-related negativity; ERN = error-related negativity; Pc = correct positivity; Pe = error positivity

For N1 amplitude the main effects of condition and congruency were not significant, *T*_WJt_/c(1.0,11.0) = 4.30, *p* = .08; *T*_WJt_/c(1.0,11.0) = 0.39, *p* = .55, respectively. The Condition x Congruency interaction was also not significant, *T*_WJt_/c(1.0,11.0) = 0.17, *p* = .68. The main effects of condition and congruency were not significant for N1 latency, *T*_WJt_/c(1.0,11.0) = 4.34, *p* = .07; *T*_WJt_/c(1.0,11.0) = 2.24, *p* = .18, respectively. The Condition x Congruency interaction was also not significant, *T*_WJt_/c(1.0,11.0) = 1.83, *p* = .20. For P2 amplitude and latency measurements, the main effects of condition and congruency and Condition x Congruency interaction were not significant (all *T* values < 2.1, all *p* values > .19). For N2 amplitude, the main effects of condition and congruency and Condition x Congruency interaction were not significant (all *T* values < 0.5, all *p* values > .52). The N2 latency similarly showed nonsignificant main effects of condition and congruency, *T*_WJt_/c(1.0,11.0) = 4.27, *p* = .07; *T*_WJt_/c(1.0,11.0) = 0.04, *p* = .85, respectively. The Condition x Congruency interaction was also not significant, *T*_WJt_/c(1.0,11.0) = 0.06, *p* = .82. For P3 amplitude and latency measurements, the main effects of condition and congruency and Condition x Congruency interaction were not significant (all *T* values < 2.4, all *p* values > .15).

For N450 amplitude, there was a significant main effect of congruency with more negative N450 amplitudes for incongruent trials compared to congruent trials, *T*_WJt_/c(1.0,11.0) = 18.17, *p* = .003. The main effect of condition was not significant, *T*_WJt_/c(1.0,11.0) = 0.07, *p* = .80. The Condition x Congruency interaction was also not significant, *T*_WJt_/c(1.0,11.0) = 0.07, *p* = .80. Analyses of N450 latency revealed no significant main effects or interactions (all *T* values < 1.1, all *p* values > .33).

The robust ANOVA on conflict SP amplitude yielded a main effect of congruency with more positive conflict SP amplitude to incongruent trials than to congruent trials *T*_WJt_/c(1.0,11.0) = 13.72, *p* = .006. There was a nonsignificant main effect of APTD versus BAL condition, *T*_WJt_/c(1.0,11.0) = 2.28, *p* = .16. The Condition x Congruency interaction was also not significant, *T*_WJt_/c(1.0,11.0) = 2.94, *p* = .12.

For response-locked ERP analyses one participant was excluded for only having two error trials in one condition. The following analyses were conducted on the remaining eleven participants. Error-trial ERN amplitude was larger (i.e., more negative) than correct-trial amplitude as indicated by a significant main effect of accuracy, *T*_WJt_/c(1.0,10.0) = 80.21, *p* < .001. The main effect of condition and the Condition x Accuracy interaction were not significant, *T*_WJt_/c(1.0,10.0) = 0.73, *p* < .41; *T*_WJt_/c(1.0,10.0) = 0.50, *p* = .52, respectively. Analyses of ERN latency yielded no significant main effects or interactions (all *T* values < 2.4, all *p* values > .16).

As expected, Pe amplitudes were larger for error trials than for correct trials; this difference was supported by a significant main effect of accuracy, *T*_WJt_/c(1.0,10.0) = 40.19, *p* < .0001. The main effect of condition and the Condition x Accuracy interaction were not statistically significant, *T*_WJt_/c(1.0,10.0)<0.01, *p* = .97; *T*_WJt_/c(1.0,10.0) = 0.04, *p* = .84, respectively, suggesting no strong relationship between the APTD procedure and response-locked ERP amplitudes.

## Discussion

The purpose of this study was to investigate the role of dopamine signaling in ACC-mediated cognitive control processes using ERPs. To this aim, an APTD method was used to reduce the neurotransmitter dopamine as a strategy for targeting the specific role of dopamine in cognitive control functions in healthy participants without using psychotropic medications. Manipulation checks confirmed the expected effects to the dopamine AA precursors, TYR/LNAA ratio, and prolactin. Specifically, there were no differences in AA levels between BAL and APTD conditions at baseline, but there were increases in all AAs during the BAL condition and a significant reduction in TYR and PHE levels in the APTD condition. Furthermore, the tyrosine/LNAA ratio was specifically decreased during the APTD condition and prolactin levels decreased in the BAL treatment but not the APTD condition, consistent with diminished hypothalamic dopamine release in the APTD condition. The data, therefore, suggest that the APTD procedure was effective in reducing dopamine synthesis in areas of the brain that include the ACC and striatum [10, 12–15]. Results should thus be interpreted in the context of an effective dopamine precursor depletion procedure.

For stimulus-locked ERPs, N450 amplitude, an index of conflict detection, was enhanced on incongruent trials compared to congruent trials, but did not significantly differ between the BAL and APTD conditions. Thus, our hypothesis of diminished N450 amplitude during dopamine precursor depletion was not supported. Results for the conflict SP ERP were similar. There was a significant differentiation between congruent and incongruent trials, but this difference did not change as a function of APTD or BAL condition. Findings indicate that dopamine precursor depletion is not effective in altering conflict detection and conflict resolution processes reflected by the N450 and conflict SP ERPs. These results were not due to potential changes in earlier sensory ERPs as the N1, N2, and P2 ERPs also did not show differences between APTD or BAL conditions.

Behavioral (RT and error rate) results also did not differ between BAL and APTD conditions regardless of task congruency. More specifically, performance on the Stroop task showed the expected increase in error rates and RTs on incongruent compared to congruent trials, but the magnitude of this difference was not larger in any specific APTD condition. These findings are in-line with the stimulus-locked ERPs and support the possibility that there are not clear differences between congruency-related conditions on the Stroop task in the current sample. These findings are somewhat in contrast to a previous study using the Stroop task and the APTD procedure. Scholes et al. [6] used a similar depletion procedure, but also included a tryptophan depletion condition to show less Stroop interference following both acute tryptophan depletion and APTD compared to BAL. A primary difference between our results and the Scholes et al. results is in the Stroop task used. Both studies used a single-trial Stroop; however, the Scholes et al. paper utilized 24 congruent trials, 24 incongruent trials, and 48 neutral trials for their Stroop task compared to 800 trials with a 75% incongruent to 25% congruent trial ratio and no neutral trials in the current study. Notably, all significant findings in the Scholes et al. paper were when incongruent trials were compared to neutral trials. Thus, there were considerable differences in conflict ratio and signal-to-noise ratio that could contribute to the differences in findings. It is possible that the APTD procedure results in differences when incongruent trials are compared to neutral trials, rather than to only congruent trials. It is also possible that the infrequent presentation of conflict (i.e., incongruent trials), done in an effort to enhance the conflict signal in the ERPs, inadvertently increased the attention to incongruent stimuli and decreased the possible subtle changes that may be present in APTD versus BAL conditions (see [17]).

For response-locked ERPs, the presence of both the ERN and Pe components was clear as evidenced by significant differences between correct trials and error trials. There were no significant differences between component amplitudes due to APTD or BAL condition. Based on the current results, there is some temptation to conclude that ERN and Pe amplitudes are not strongly dependent on dopamine given the current lack of significant between-condition differences. However, we do not feel that such an interpretation is currently warranted. As noted above, pharmacological and pathology studies suggest there is a role of dopamine, at least for the ERN. Thus, we feel future replication studies are needed prior to making strong conclusions regarding the APTD method and error-related ERPs. Furthermore, it is possible that a more automatic conflict task with fewer response options, such as an arrow flanker task, might contribute to different results. Indeed, the previous studies examining the ERN and Pe using pharmacological manipulations of dopamine in healthy individuals have primarily used flanker tasks [2, 3, 54, 55]. The number of response options (four in the current task versus two in the flanker), the requirement to read color-words that may be less automatic (e.g., the inclusion of gray in the current task), and the generally slower responses in Stroop versus flanker tasks could be contributory to the current findings. In total, our results do not preclude the role of dopamine in cognitive control and performance monitoring processes, but may suggest that pharmacological manipulations for dopamine-related hypothesis testing are more robust.

Positron emission tomography studies of striatal dopamine release [13] and neuroendocrine studies of prolactin release [61, 87] suggest that APTD procedures reduce striatal and hypothalamic dopamine release by approximately 30 to 50%. Larger effects can be seen under challenge conditions [10, 14]. These decreases lead to some behavioral effects quite consistently, such as decreased motivation to sustain effort to obtain pharmacological [88, 89] and monetary rewards [90]. In comparison, APTD does not consistently affect other behaviors, such as spatial recognition or working memory performance [61, 91–95], or lead to Parkinsonian symptoms, a side effect that typically emerges only after dopamine reductions of more than 80%. This lack of Parkinsonian symptoms and modest neurocognitive decrements reduces confounding effects for some research purposes but seems a limitation for others. We therefore conclude only that the APTD procedure is not associated with ERN or Pe changes in the current sample, not that dopamine is uninvolved in the generation of these components.

Only one other ERP study we are aware of showed an effect of the APTD procedure on ERPs. Specifically, Linssen et al. [96] showed no behavioral working memory changes on a Sternberg task, but showed some alterations in latency and/or amplitude of the P150, N200, and P3b components of the ERP. One resolution to these discrepant findings may be that there are individual differences in the magnitude of dopamine decrease following APTD. Indeed, in a combined PET–neurocognitive task study, the greater the decrease in dopamine release, the greater the changes in spatial working memory and accuracy [95].

The use of the APTD procedure is novel in this study; however, disentangling contributions to phasic *versus* tonic dopamine release is difficult. Tyrosine depletion decreases dopamine release in response to stimulation that simulates physiologically relevant burst firing [15]. These effects have been demonstrated in striatum and medial prefrontal cortex [15]. More substantial decreases in dopamine synthesis and release can be achieved with the tyrosine hydroxylase inhibitor, alpha-methyl-para-tyrosine (AMPT). Whereas the effects of APTD are preferential for dopamine [14, 15, 97, 98], AMPT affects dopamine and norepinephrine equally [98]. Moreover, the larger effects of AMPT can induce Parkinsonian-like symptoms that might confound the measures of interest here. Dopamine receptor ligands can also be used. Low dose agonists are thought to preferentially bind to high-affinity autoreceptors, but selecting the right dose is difficult and likely susceptible to marked individual differences. Dopamine receptor antagonists either bind to many non-dopamine receptors or are selective for D2 receptors only, resulting in effects that may reflect disproportionate activation of D1 receptors. The limitations of each of these methods suggest that the fullest understanding of the role of dopamine will be best achieved by systematically applying all of these approaches. Here, we have started with APTD, a method that decreases pre-synaptic dopamine availability and the stimulation of all dopamine receptors.

The absence of significant differences between the APTD and BAL conditions across ERPs in the current study should be interpreted in light of the following limitations. First, we only included males in the sample due to previous findings showing sex-related differences in conflict and cognitive control processes, as well as to remove possible effects of menstrual phase and diurnal estrogen variation on dopamine levels [63, 64, 99, 100]. Second, the sample size is small. The expense and time taken for the within-groups design was a contributing factor; however, the size of the sample is similar to several other APTD studies (for example, [6, 12, 101]) and the number of people recruited and run through the paradigm would need to be so large (> 200) to detect between-condition differences (given the very small differences we found) that it would not be feasible to complete given the expense and difficulty of the APTD procedure. Thus, it is unlikely that researchers will undertake the time and expense to gather such a large sample using this procedure. Third, the APTD process has some side effects and limitations. Some participants became somewhat nauseated during both the BAL and APTD that may have decreased attention to study stimuli, although rates of nausea did not differ between conditions (5 total reported difficulties, 3 during the placebo condition). Fourth, there were no direct measures of dopamine in the brain. Inferences of dopamine levels were made from reliable and frequently used precursors, but not a direct measure. Finally, there was no effect of APTD on behavioral RT and accuracy data. The absence of significance on the behavioral results may have been due to the small sample size, although there is not a strong correspondence between behavioral and ERP-related cognitive control findings [16, 102].

Strengths of the study include a good experimental control with a randomized, within-subjects, double-blind, placebo-controlled design, the presence of the hypothesized changes in dopamine precursor proteins indicating the dopamine manipulation was likely effective, and robust and stringent statistical analyses to ensure findings are not reported that are due to outliers or unmet assumptions of traditional statistical analyses.

### Conclusions

We conclude that acute dopamine precursor depletion does not strongly affect ERP manifestations of conflict detection, conflict resolution, or performance monitoring processes. In the absence of very large samples and investment of resources, we suggest future studies rely upon more robust dopamine medications or pathology in the study of the possible role of dopamine in cognitive control and related ERPs.

## Supporting Information

S1 Dataset Pla = Placebo (i.e., balanced condition); Dep = Depletion (i.e., APTD condition); Pre = before administration of the balanced or APTD mixture; Post = after balanced or APTD condition; Tyr = tyrosine; Phe = phenylalanine; Val = valine; Trypt = tryptophan; Isoleu = isoleucine; Leu = leucine; Cong = congruent; Inco = incongruent; RT = response time; ER = error rate. (CSV)
